# Prevalence and complete genome of bovine norovirus with novel VP1 genotype in calves in China

**DOI:** 10.1038/s41598-019-48569-4

**Published:** 2019-08-19

**Authors:** Yuelin Wang, Hua Yue, Cheng Tang

**Affiliations:** 1 College of Life Science and Technology, Southwest Minzu University, Chengdu, China; 2 Key Laboratory of Qinghai–Tibetan Plateau Animal Genetic Resource Reservation and Utilization, Chengdu, China

**Keywords:** Molecular biology, Whole genome amplification, Genotype

## Abstract

Bovine norovirus (BNoV) is a diarrhea-causing pathogen of calves. In this study, 211 diarrheic fecal samples were collected from 25 farms across six provinces in China, between November 2017 and September 2018. 20.4% of the samples were detected as BNoV-positive by RT-PCR. Phylogenetic analyses based on RdRp, VP1, and VP2 fragments revealed these BNoV strains had unique evolutionary characteristics. The complete genome of strain Bo/BET–17/18/CH was successfully sequenced. It was 7321 nucleotides (nt) in length, shared 79.4–80.9% nt identity with all five BNoV genomes, clustered on a separate branch of the phylogenetic tree, suggesting that strain Bo/BET–17/18/CH could represent a novel BNoV strain. Two interesting characteristics were found in the genome: (i) the VP1 sequence differed greatly from known BNoV VP1 sequences; (ii) a recombination event is predicted within the ORF1–ORF2 overlap. Moreover 16.3% (7/43) of the BNoV were identified as the novel VP1 genotype, which were distributed on four farms across two provinces, indicating that the novel VP1 genotype strain has spread. To our knowledge, this is first description of the molecular and genomic characteristics of BNoV in China. These findings extend our understanding of the genetic evolution and epidemics of BNoV.

## Introduction

Noroviruses (NoV) are important pathogens causing gastroenteritis in children and young animals. These viruses can be classified into at least six distinct genogroups (GI–GVI) based on their major capsid protein (VP1) sequences^1^. The bovine noroviruses (BNoV) cluster into genogroup III (GIII), and can be further divided into two distinct genotypes: genotype 1 (GIII.1) and genotype 2 (GIII.2). The GIII.1 strains appear more virulent than the GIII.2 strains^2,3^. Because there is no suitable cell culture system for BNoV, the precise pathogenicity of BNoV still requires investigation^4^. Currently, GIII.1 strains have been detected in 9 countries, the GIII.2 strains are the most prevalent worldwide, and have been detected in 16 countries^5–7^. In 2009, a NoV strain identified in sheep feces was proposed to be NoV GIII.3 based on the complete capsid genes of the strain^8^. However, there is only one complete VP1 sequence for this genotype in the GenBank database (GenBank accession number EU193658), and no genome information for this strain is yet available.

Currently, five complete genome sequences of BNoV are available in GenBank, one from a GIII.1 strain (GenBank accession number AJ011099) and four from GIII.2 strains (GenBank accession number EU794907, JX145650, AF097917 and AY126474). The BNoV genomes are organized into three open reading frames (ORF). Starting from the 5′ end of the genome, ORF1 encodes the viral nonstructural proteins, including RNA-dependent RNA polymerase (RdRp). RdRp is a key enzyme, responsible for transcription, the replication of the viral genome, and the accurate initiation of RNA synthesis, which is essential for preventing the loss of viral genetic information^9,10^. ORF2 encodes VP1, which is the major structural component of the NoV and is involved in receptor recognition, host specificity, and immunogenicity^11^. The X-ray crystallographic structure of human NoV shows that VP1 contains two major domains, a well-conserved shell (S) domain and a more variable protruding (P) domain. The P domain is further divided into the P1 and the highly variable P2 domains^12^. The X-ray crystallographic structure of the BNoV P domain indicates that it is similar to the GI.1 P domain, whereas the P1 interface loop is very similar to that of GII^13^. Variations in the VP1 amino acid (aa) sequence occur frequently, and may allow the virus to escape antibody neutralization, these variation are also associated with the emergence of new epidemic strains^14,15^. ORF3 is a hypervariable region in the NoV and encodes the minor capsid protein (VP2), which may function in maintaining the stability of NoV particles^16^.

Recently, our team confirmed the presence of BNoV in China^7^, but the prevalence and molecular characteristics of BNoV in China are still unclear. In this study, we collected new samples of calf diarrheic feces from six provinces to investigate the molecular prevalence and genomic characteristics of BNoV in China. Unexpectedly, a potentially novel BNoV strain was identified based on genome sequences, and this novel genotype has spread in local provinces.

## Results

### BNoV detection and co-infections

**Figure 1:**
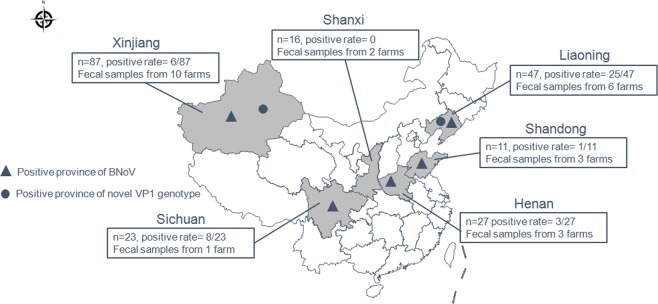
Number of calves from 6 provinces, China, 2017–2018. The six provinces that were sampled were indicated in grey. n values indicate the total number of samples in each province; positive rate indicate the BNoV positive rate.

20.4% (43/211) of the diarrheic samples were detected as BNoV-positive by RT-PCR, and 14 of the 25 farms sampled in five provinces were positive for BNoV (Fig. 1). Of the 43 BNoV-positive diarrheic samples, 38 were co-infected with bovine rotavirus (BRV), Bovine coronavirus (BCoV), and/or Bovine viral diarrhea virus (BVDV) (Table 1). The co-infected of distinct BNoV genotype in each region was shown in Supplementary Table 3.

**Table 1:** The enteric pathogens co–infected with BNoV.

Enteric pathogens	The positive rate (%)
BNoV (only)	11.6
BNoV + BRV	9.3
BNoV + BRV + BCoV	46.6
BNoV + BRV + BVDV	11.6
BNoV + BCoV + BVDV	2.3
BNoV + BRV + BCoV + BVDV	18.6

### Phylogenetic analyses of RdRp, VP1, and VP2 genes

**Figure 2:**
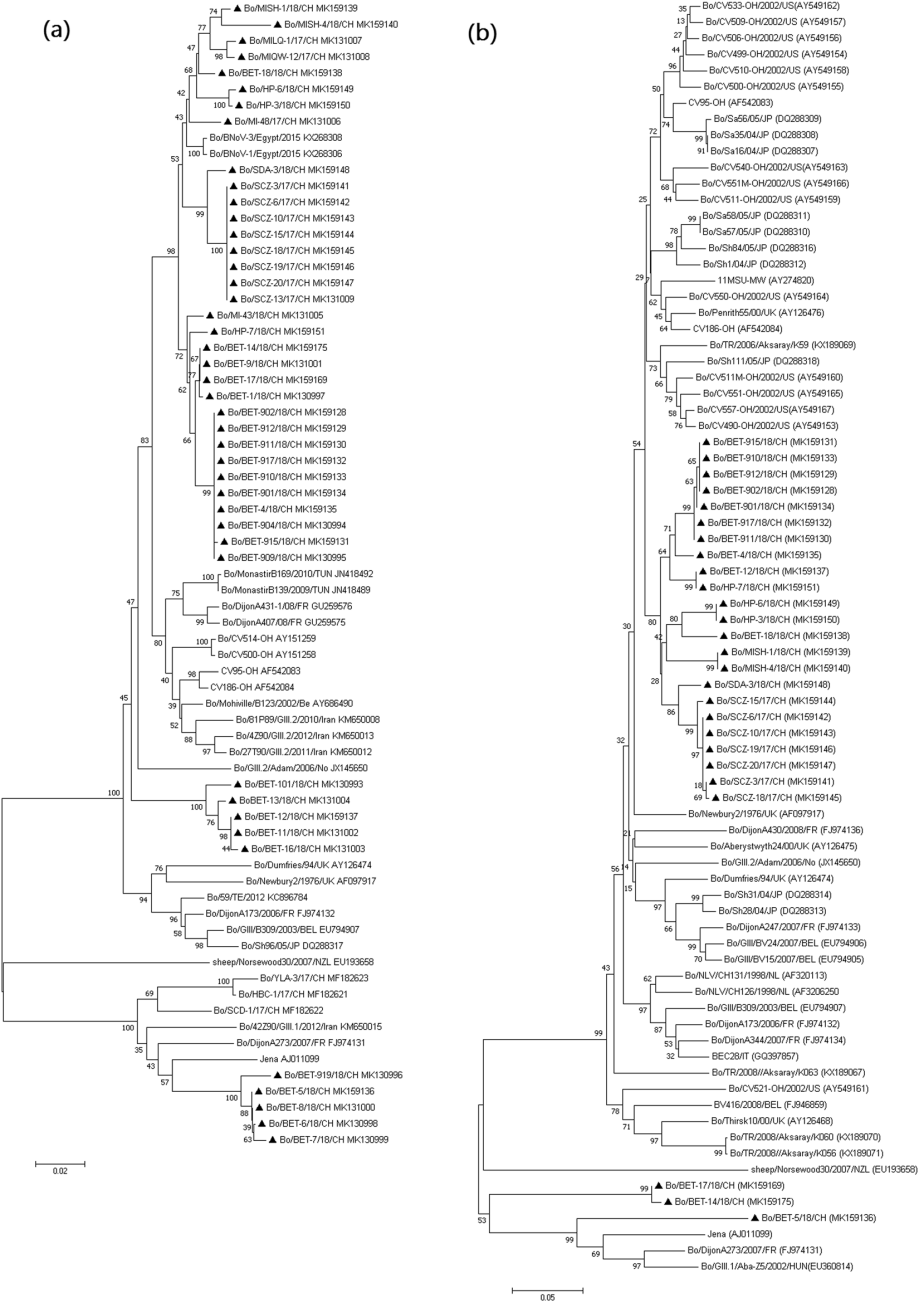
Phylogenetic trees based on the RdRp fragments (**a**) and 505 bp sequence of the VP1 fragments (**b**). Sequence alignments and clustering were performed by ClustalW in MEGA 7.0 software. The tree was constructed by the neighbor–joining method with bootstrap values calculated for 1000 replicates. The strains in this study were marked with triangle.

All 43 RdRp fragments were sequenced and the results confirmed that all the fragments belonged to BNoV. A neighbor-joining phylogenetic tree based on the RdRp fragments indicated that 38 of the strains from this study clustered in GIII.2 and five clustered in GIII.1 (Fig. 2a). Among the 38 GIII.2 strains, 33 clustered on a large branch with two Egyptian strains (GenBank accession numbers KX268308 and KX268306), and the remaining five strains clustered on an independent branch.

Twenty-six fragments of VP1 (505 bp) from seven BNoV-positive farms across five provinces were successfully sequenced. A neighbor-joining phylogenetic tree based on all the available sequences of VP1 that were ≥505 bp indicated that 23 of the BNoV strains from this study clustered with GIII.2, one strain clustered with GIII.1, and the remaining two strains clustered on a unique branch. All 23 GIII.2 strains clustered on an independent branch of the phylogenetic tree (Fig. 2b) and shared 92.1–100% nucleotides (nt) identity (97.6–100% aa identity) with each other, and 86–94.3% nt identity (95.3–100% aa identity) with the GIII.2 strains in the GenBank database. The strain that clustered with GIII.1 shared 81.4–85.4% nt identity (91.7–92.9% aa identity) with the group of GIII.1 strains.

Interestingly, VP1 of two strains (GenBank accession numbers MK159175 and MK159169) clustered on a unique branch and shared 99.4% nt identity (98.8% aa identity) with each other, but only 78.3–80.4% nt identity (87–88.8% aa identity) with all known VP1 genes of GIII.1 strains, 77.1–81% nt identity (82.2–84.6% aa identity) with all known VP1 genes of GIII.2 strains (including the strains of this study), and 75.9–76.5% nt identity (81.1–82.2% aa identity) with the VP1 gene of the single possible GIII.3 strain.

**Figure 3:**
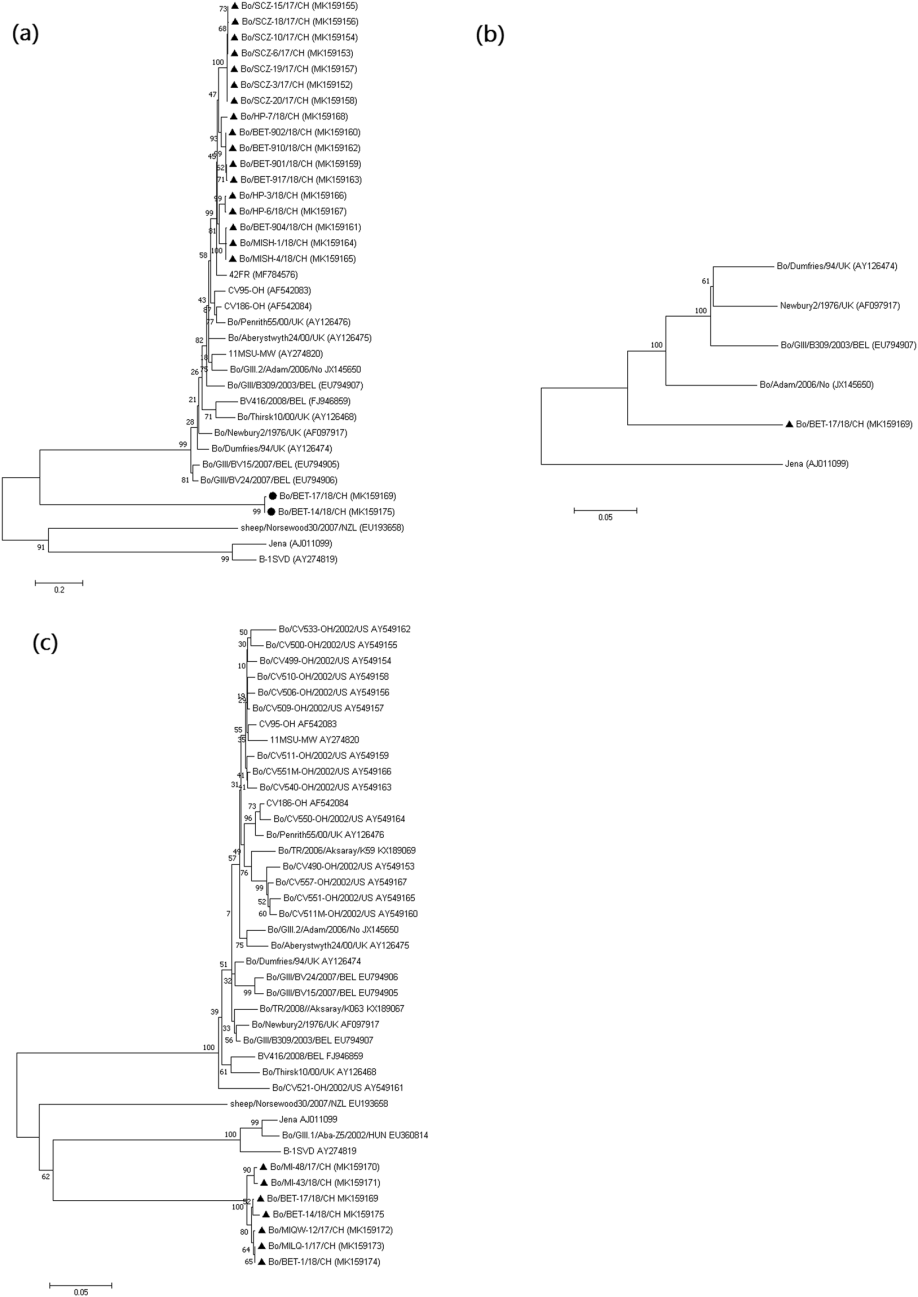
Phylogenetic tree based on the 803 bp sequence of VP2 fragments (**a**), whole genome (**b**), complete VP1 amino acid sequences (**c**). Sequence alignments and clustering were performed by ClustalW in MEGA 7.0 software. The tree was constructed by the neighbor–joining method with bootstrap values calculated for 1000 replicates. The strains in this study were marked with triangle, and two strains from novel genotype were marked with circle in the Phylogenetic tree of VP2.

Seventeen fragments of VP2 (803 bp) were successfully amplified from the five BNoV-positive farms across four provinces. In a neighbor-joining phylogenetic tree based on all the available sequences of the BNoV VP2 gene that were ≥803 bp, all 17 strains clustered on a large branch with Egyptian strain MF784576 (Fig. 3a). The 17 strains from this study and the Egyptian strain shared 92.4–100% nt identity (92.7–100% aa identity) with each other, and 61.5–89.8% nt identity (56.9–91.3% aa identity) with other GIII strains in the GenBank database. Interestingly, the 17 strains from this study and the Egyptian strain had three identical amino acid mutations (G73E, D78G, and S148T) in the VP2 region compared with the VP2 sequences available in the GenBank database.

### Genomic characterization of strain Bo/BET–17/18/CH

Because the VP1 genes of strains Bo/BET-17/18/CH and Bo/BET14/18/CH showed significant differences compared from all other GIII VP1 genes, strain Bo/BET-17/18/CH was selected for genomic amplification. The complete genome of strain Bo/BET–17/18/CH was successfully sequenced (GenBank accession number MK159169). The genomic sequence of strain Bo/BET-17/18/CH was verified by 2 different sets of primers (Supplementary Table 2), which shared 100% nt identity. The linear genome is 7321 nt in length, with a G + C content of 57.6%, including a 21nt 5′ untranslated region (UTR) and a 57nt 3′ UTR. ORF1 is located at nt 22–5076 and encodes 1684 amino acids, which form nonstructural proteins. ORF2 is located at nt 5063–6637 and encodes 524 amino acids, which form VP1. ORF3 is located at nt 6627–7265 and encodes 212 amino acids, which form VP2.

The full-length genome of strain Bo/BET–17/18/CH shares 79.4–80.9% nt identity with four complete genomes of GIII.2 strains and 70.7% nt identity with the only complete genome of GIII.1 in the GenBank database. The aa identities of the non-structural proteins and structural proteins of strain Bo/BET–17/18/CH with those of five other GIII strains are shown in Table 2.

**Table 2:** Amino acid identities of strain Bo/BET17/2018/CH compared with 5 GIII genomes.

Strain	Amino acid identities (%)								
	ORF1						VP1		VP2
	P48	NTPase	P22	VPg	Pro	RdRp	Complete	P2	
Newbury2	91.5	98.1	96.3	96.8	98.9	97.8	71.2	53.5	66.8
B309	88.8	97.8	96.3	94.4	98.9	97.8	70.7	54.3	67.4
Adam	93.3	97.8	95.2	95.2	97.2	99.0	69.5	52.0	66.4
Dumfries	90.6	98.3	96.3	93.6	98.9	97.8	70.7	54.3	66.8
Jena	75.7	91.7	68.6	78.4	88.4	89.7	71.4	48.4	60.7

On a neighbor-joining phylogenetic tree based on the complete genomes of BNoV, strain Bo/BET–17/18/CH was located on an independent branch, indicating that it may represent a novel BNoV strain (Fig. 3b). Phylogenetic analyses based on P48, NTPase, P22, VPg, Pro, and RdRp amino acid sequences showed that strain Bo/BET–17/18/CH is most closely related to GIII.2 strains (data not shown).

A neighbor-joining phylogenetic tree was constructed based on the complete GIII VP1 amino acid sequences available in the GenBank database and the 2 novel VP1 sequences as well as 5 novel VP1 sequences subsequently obtained in this study. On the resulting tree, the seven strains from this study clustered on a separate large branch (Fig. 3c).

The VP1 proteins of strains Bo/BET–17/18/CH and Bo/BET–14/18/CH are 524 aa long and share an identical amino acid organization. They are five aa longer than the VP1 proteins of the GIII.1 strains and two aa longer than those of the GIII.2 strains. The VP1 proteins of Bo/BET–17/18/CH and Bo/BET–14/18/CH share 99.7% aa identity with each other, 70.3–71.4% aa identity (68.6–69.4% nt identity) with the group of GIII.1 strains, 69–71.2% aa identity (66.2–68.1% nt identity) with the group of GIII.2 strains, and 71.2–71.6% aa identity (68.7–68.9% nt identity) with the potential GIII.3 strain in the GenBank database. Based on VP1, strain Bo/BET–17/18/CH showed a pairwise distance of 33.1% to the GIII.1 prototype strain Jena, 37.3% to the GIII.2 prototype strain Newbury2, and 32.6% to the potential GIII.3 strain in the GenBank database.

An amino acid alignment of all the complete GIII VP1 sequences in the GenBank database showed that strains Bo/BET–17/18/CH and Bo/BET–14/18/CH share 63 identical mutations in the 524 aa of VP1: 7 mutations in the 176 aa of the conserved S domain and 56 mutations in the 297 aa of the P domain, 35 of which occur in the 127 aa of the P2 domain.

**Figure 4:**
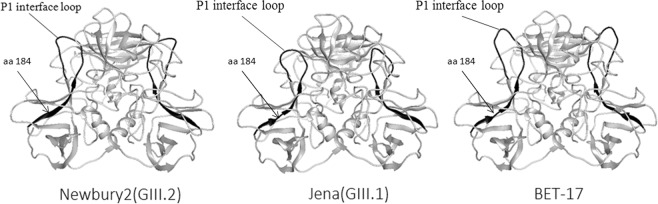
Three dimensional modeling of P domain (strains Newbury2, Jena and BET-17). The structures were based on homology modeling (PDB ID 5e6t) for the three strains. The variation region compared to the reference strain is marked in black. The BET-17 contained similar structural elements as GIII.1, but has a longer interface-loop.

The three-dimensional model of strain Bo/BET-17/18/CH based on the crystal structure of BNoV P domain showed that Bo/BET-17/18/CH VP1 contains structural elements similar to those of Jena (GIII.1) in the P domain, in which the β-sheet present at aa 184 in Newbury2 (GIII.2) becomes a random coil. Moreover, in Bo/BET–17/18/CH P domain, the interface loop is 2 aa longer than that in strain Newbury2 (GIII.2) and 4 aa longer than that in strain Jena (GIII.1) (Fig. 4).

**Figure 5:**
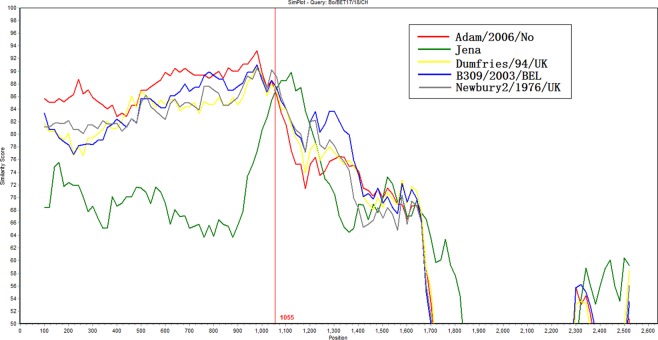
The SimPlot analysis of strain Bo/BET–17/18/CHA nucleotide (nt) identify plot comparing a 2637 bp fragment (1–1076 bp belonging to RdRp and 1063–2637 bp belonging to complete VP1) of strain Bo/BET–17/18/CH with BNoV strains Newbury2, B309, Adam, Dumfries Jena is shown. The putative recombination breakpoint is located at the 1055 bp. The vertical axis indicates the similarity (%) of nucleotide sequences between the query strain and other reference strains. The horizontal axis indicates the nucleotide positions. SimPlot analysis was performed using a window size of 200 nt and step size of 20 nt.

Recombination events were predicted with SimPlot and Recombination Detection Program (RDP) 4.0 with RDP, GeneConv, Chimaera, MaxChi, SiScan, and BootScan (recombinant score: 0.548). A recombination breakpoint was predicted at nt 5034 in the genome. The putative major parental strain was Bo/Adam/2006/No (GenBank accession number JX145650), but the minor parental strain was not found. However, using SimPlot, recombination breakpoint was predicted at 5055 bp in genome (Fig. 5). Although the recombination breakpoints predicted by RDP 4.0 and SimPlot differ, both programs showed that the recombination breakpoint was located at the ORF1–ORF2 overlap. A phylogenetic tree based on the sequences upstream and downstream from the predicted recombination breakpoint supported a recombination event (data not shown).

### Screening for novel VP1 genotypes

In addition to strains Bo/BET–17/18/CH and Bo/BET–14/18/CH, 5 strains of the BNoV-positive sample were screened as the potential novel VP1 genotype based 467 bp VP1 fragments by RT–PCR. Furthermore, the 5 complete VP1 genes were successfully amplified from samples (GenBank accession numbers MK159170-MK159174). Therefore, a total of 7 novel VP1 complete sequences were obtained in this study. Of the 7 samples containing BNoV with novel VP1, 2 samples were detected as only BNoV positive; 3 samples were detected as co-infected with BRV and BCoV; 1 sample was detected as co-infected with BRV and BVDV; 1 sample was detected as co-infected with all three viruses.

A neighbor-joining phylogenetic tree based on 7 complete novel VP1 sequences and all the complete VP1 sequences available in the GenBank showed that the 7 strains clustered on a separate branch (Fig. 3c). The 7 novel complete VP1 sequences shared 97.2–99.9% nt identity (98.1–100% aa identity) with each other, 68.5–69.4% nt identity (70.9–73.0% aa identity) with the GIII.1 strains, 66.2–68.2% nt identity (69.8–71.9% aa identity) with the GIII.2 strains, and 69.2–70.1% nt identity (73.4–73.8% aa identity) with the potential GIII.3 strain in the GenBank. Moreover, the 7 novel VP1sequences shared 61 identical aa mutations out of 524 aa VP1 proteins, compared with all 34 known complete GIII VP1 sequences.

### Nucleotide sequence accession numbers

All the sequencing results in this study have been submitted to the GenBank database under accession numbers MK130993–MK131009 and MK159128–MK159175.

## Discussion

In this study, BNoV was detected with RT–PCR in 43 of 211 (20.4%) diarrheic samples, with an extensive geographic distribution, and GIII.2 was the dominant genotype. These results show that BNoV is circulating widely in calves in China. Co-infection of BNoV with BRV, BCoV, and/or BVDV is very common, which is similar to the situation reported in South Korea^17^. These co-infections will require more complex diagnoses and will make the clinical treatment of calf diarrhea more difficult.

Interestingly, phylogenetic analyses of RdRp, VP1, and VP2 gene fragments indicated that most of the sequences obtained from distinct geographic regions of China in this study were closely related, and did not cluster into distinct branches based on their geographic origins. This indicates that the Chinese BNoV strains have unique evolutionary characteristics. Similar phenomena have been recently reported for human NoV (GI and GII) in China^18^.

Bo/BET–17/18/CH is the first complete genome of BNoV determined in China. In a phylogenetic analysis of strain Bo/BET–17/18/CH and the five available BNoV genomes, strain Bo/BET–17/18/CH clustered on a separate branch and shared 70.7% nt identity with the only GIII.1 genome and 79.4–80.8% nt identity with the four GIII.2 genomes. The Bo/BET–17/18/CH genome has two significant characteristics: VP1 may represent a novel VP1 genotype, because it is distinct from all other GIII VP1 proteins; and a recombination event is predicted to have occurred in the genome. These characteristics suggesting that it may represent a novel BNoV strain.

A previous study suggested that in the classification of NoV based on complete VP1 amino acid sequences, a new genotype of NoV requires a pairwise distance of 14.3–43.8% from the other genotypes, and a new genogroup requires a pairwise distance of 44.9–61.4% from the other genogroups^19^. This method has been used extensively in the classification of NoV^1,20,21^. An analysis of all 34 complete GIII VP1 sequences available in the GenBank database, including three GIII.1 strains, 30 GIII.2 strains, and one possible GIII.3 strain, showed that strains of the same genotype share ≥91.6% aa identity, whereas distinct VP1 genotypes share only 67.4–73.0% aa identity. The VP1 proteins of strains Bo/BET–17/18/CH and Bo/BET–14/18/CH from this study share 70.4–74.1% aa identity with all 34 complete GIII VP1 sequences, and the pairwise distances between the two complete VP1 sequences (Bo/BET–17/18/CH and Bo/BET–14/18/CH) determined in this study and all 34 complete VP1 sequences were 32.6–39.5%. A phylogenetic tree based on the deduced amino acid sequences of the complete capsid region showed that strains Bo/BET–17/18/CH and Bo/BET–14/18/CH clustered on a unique branch (Fig. 3c). These results suggest that strain Bo/BET–17/18/CH represents a novel VP1 genotype in the GIII group, which we propose should be designated ‘GIII.4’.

The VP1 protein of NoV is involved in self-assembly and capsid formation, receptor recognition, host specificity, antigenic diversity, and immunogenicity^11^. The P2 domain and P1 interface loop are involved in the binding of ABO histo-blood group antigens (HBGAs), which are associated with its hosts’ susceptibility to NoV infection^22,23^. Different NoV VP1 genotypes may have different abilities to bind HBGAs. For example, the GII.4 viruses are thought to bind a wider range of HBGAs and therefore have larger susceptible populations than the other genotypes^24^. A three-dimensional model was generated based on the X-ray crystallographic structure of the BNoV VP1 P domain and showed that Bo/BET–17/18/CH was most similar to that of strain Jena (GIII.1), although it has a longer interface loop (Fig. 4). Strain Bo/BET–17/18/CH also has many amino acid mutations in VP1, especially in the P2 domain. These variations in VP1 may have implications for the interaction between the novel VP1 genotype and HBGAs. The interactions between the different VP1 genotypes and HBGAs warrant further study.

A recombination event was observed in strain Bo/BET–17/18/CH, and the breakpoint was located in the ORF1–ORF2 overlap. The major parent was strain Bo/Adam/2006/No (GenBank accession number JX145650), but the minor parent was not found. Similar phenomena have been reported in human NoV and porcine NoV^25–27^, which may be attributable to the limited number of NoV sequences available. RNA recombination is considered to be one of the main drivers of NoV evolution^24^. The majority of intergenotype and intragenotype recombination events in NoV, including BNoV, occur at a single location in the ORF1–ORF2 overlap^28,29^. Recombination at this junction allows the virus to alter its viral capsid while retaining the region involved in the replication of the viral genome. This reorganization can also allow the virus to escape herd immunity^24^. As an example, the recombinant type GII.Pb/GII.3 is one of the most prevalent NoV, and still prevalent today in children^30^. Until now, two recombinant types have been identified in BNoV: one is GIII.2-related RdRp sequence and a GIII.1-related capsid sequence, and the other is GIII.1-related RdRp sequence and a GIII.2-related capsid sequence. The second recombinant type is circulating in different geographic regions^31–35^. However, the first recombinant type has only been reported in the United States^34^. Interestingly, strain Bo/BET–17/18/CH is the strain of a recombination event involving a GIII.2-related RdRp sequence and a novel genotype-related capsid sequence. The prevalence of the novel strain must be monitored further.

VP1 of strain Bo/BET–17/18/CH is significantly different from those of all other GIII strains, including in its conserved S domain. 16.3% (7/43) of our BNoV-positive samples were identified as the novel VP1 genotype based on complete VP1 sequences. These seven strains were from four farms across two provinces that are more than 3000 km apart, indicating that this new genotype has spread across local provinces (Fig. 1). Interestingly, on the farms positive for the novel VP1 genotype, the proportions of the novel genotype in the BNoV-positive samples were 1/1, 1/2, 2/2, and 3/4. Therefore, it seems that the novel VP1 genotype strains were predominant on the affected farms. However, this result may be biased because the number of samples tested was small.

In conclusion, in this study, we have demonstrated that BNoV is circulating wildly in diarrheic calves in China, and that the RdRp, VP1, and VP2 genes of the virus show unique evolutionary. A BNoV strain with a novel VP1 genotype was identified based on its complete genome. Notably, this strain has spread in local provinces in China. To the best of our knowledge, this is the first report of the molecular and genomic characteristics of BNoV in China. These findings will extend our understanding of the prevalence and genetic evolution of BNoV.

## Methods

### Sample collection

A total of 211 diarrheic fecal samples were collected from calves ( ≤ 3 months of age) on 25 farms across six provinces of China (Fig. 1) between November 2017 and September 2018. The samples were shipped on ice and stored in sterile 50 ml centrifuge tubes at −80 °C.

### RNA extraction and cDNA synthesis

The fecal samples were fully resuspended in phosphate-buffered saline (1:5) and the total RNA in 400 μL of the fecal suspensions was extracted with Trizol™ Reagent (TaKaRa, China), according to the manufacturer’s instructions. cDNA was synthesized with the PrimeScript™ RT–PCR Kit (TaKaRa) and stored at −20 °C until required.

### Detection of BNoV

BNoV was detected with RT–PCR targeting a 532 bp fragment of the RdRp gene, according to a previous report^36^. All the RT–PCR products were sequenced in both directions.

### Screening for co-infection with major bovine enteric pathogens

To investigate the co-infection of the calves with BRV, BCoV, and BVDV, all BNoV-positive diarrheic samples were subjected to specific RT–PCR assays for these viruses. BRV and BCoV were detected according to our previous report^37^, and BVDV according to a previous study^38^.

### Amplification of partial VP1 and VP2 genes

A pair of degenerate primers was designed to amplify the partial RdRp gene and the 5′ end of VP1 (located at nt 4868–5567 in reference genome Bo/Newbury2/1976/UK, 700 bp in length), which contained 505 bp of VP1, based on genome sequences available in GenBank. The primer sequences were BNV–F1, 5′-TGCTTTGCCTCCTCGGCG-3′ and BNV–R1, 5′-CCTGRTGGAARAGGATGTT-3′.

A pair of primers was designed to amplify the VP2 fragment (located at nt 6441–7243 in reference genome Bo/Newbury2/1976/UK, 803 bp in length), based on genome sequences available in GenBank. The primer sequences were BNV–F2, 5′-GTCGGGTCCTGTTCGAGG-3′ and BNV–R2, 5′-AATGGCATCCGATCTGTATT-3′. All amplification products were sequenced in both directions.

### Complete genome amplification of strain Bo/BET–17/18/CH

Nine pairs of primers were used to amplify the genomic sequence of BNoV strain Bo/BET–17/18/CH, including the 5′ end of the genome (Supplementary Table 1). Moreover, the complete genomic sequence of Bo/BET-17/18/CH was verified by using the new 9 pairs of primers which target distinct sites (Supplementary Table 2), among which one pair of primers (BNV-F18/R18) is used to amplify the fragment spanning the predicted recombination breakpoint. The 3′ end of the complete viral genome was obtained with the rapid amplification of cDNA ends (RACE) using the Smart™ RACE cDNA Amplification Kit (Clontech, Palo Alto, CA, USA). All the PCR products were purified with the Omega Gel kit (Omega, USA), according to the manufacturer’s instructions. They were then cloned into the pMD19-T Vector (TaKaRa Bio Inc.) and used to transform competent *Escherichia coli* DH5α cells (Yeasen, China) for sequencing. Two pairs of identical primers were used to amplify the complete VP1 sequence of strain Bo/BET–14/18/CH in this study.

### Screening for novel VP1 genotypes in BNoV-positive samples

To investigate the prevalence of the novel VP1 genotype, a pair of primers was firstly designed to screen potential novel VP1 sequences with 467 bp (located at nt 5240–5706 in reference genome Bo/BET–17/18/CH; GenBank accession number MK159169), the primer sequences were BNV–S–F, 5′-CCCCAGGGTGAGTTTACG-3′ and BNV–S–R, 5′-GGAGGGACCAGGAAGAAGA-3′. Then, two pairs of primers were designed to amplify the complete novel VP1 gene from target samples according to the genome Bo/BET–17/18/CH. The primer sequences were as follows: BNV-cap-F1: 5′-ACGACGATCCGAGTGAAA-3′ and BNV-cap-R1: 5′-AGTTGTGGTCCCAAGAAGC-3′ (the amplicon product is 1068 bp long and located at nt 4817–5884 in reference genome Bo/BET–17/18/CH); and BNV-cap-F2: 5′-CCAATCTGCCAATCTCGG-3′ and BNV-cap-R2: 5′-GGGCCATTCCAATCAAGC-3′(the amplicon product is 1181 bp long and located at nt 5742–6922 in reference genome Bo/BET–17/18/CH).

All PCR products were purified with the Omega Gel kit (Omega), according to the manufacturer’s instructions. They were then cloned into the pMD19-T Vector (TaKaRa Bio Inc.) and used to transform competent *E. coli* DH5α cells (Yeasen) for sequencing.

### Sequence, phylogenic, and recombination analyses, and molecular modeling

The sequences were assembled with the SeqMan software (version 7.0; DNASTAR Inc., WI, USA). To analyze the genome organization, the putative ORFs and their corresponding amino acids were predicted with the ORF Finder tool (http://www.ncbi.nlm.nih.gov/gorf/gorf.html). The nucleotide and deduced amino acid sequence homologies were determined with the MegAlign program of the DNASTAR 7.0 software (DNASTAR Inc). Pairwise distances between the amino acid sequences of VP1 (with gaps) were calculated with the Jones–Taylor–Thornton model in the MEGA 7.0 software. Phylogenetic trees were constructed with the neighbor-joining method in the MEGA 7.0 software, with a bootstrap analysis of 1000 replicates. Recombination events were assessed with the SimPlot software (version 3.5.1) and the RDP (version 4.0) with the RDP, GeneConv, Chimaera, MaxChi, BootScan, SiScan, and 3Seq methods. A three-dimensional model of the Bo/BET-17/18/CH capsid P domain was generated at the Swiss-Model server (http://swissmodel.expasy.org/) from the crystal structure of the GIII capsid P domain (PDB ID 5e6t)^13^, and the software program Chimera (available at http://www.cgl.ucsf.edu/chimera) was used to visualize it.

### Compliance with ethical standards

This study did not involve animal experiments other than the fecal sampling of diarrheic calves when farms were visited for clinical treatment.

## Supplementary information

Supplementary information
