# Supplementary material for: Prevalence and complete genome of bovine norovirus with novel VP1 genotype in calves in China
Source: Sci Rep. 2019 Aug 19;9:12023. doi: 10.1038/s41598-019-48569-4 (PMC6700072; doi:10.1038/s41598-019-48569-4)
Supplement: Supplementary file 1 — Supplementary information [file 41598_2019_48569_MOESM1_ESM.pdf]

Prevalence and complete genome of bovine norovirus with novel VP1 genotype in calves in China

Yuelin Wang[1], Hua Yue[1, 2]*, Cheng Tang[1, 2]*

[1] College of Life Science and Technology, Southwest Minzu University, Chengdu, China

[2] Key Laboratory of Qinghai–Tibetan Plateau Animal Genetic Resource Reservation and Utilization, Chengdu, China

Correspondence and requests for material should be addressed to C.T. (email: tangcheng101@163.com) and H.Y. (email: yhua900@163.com)

Supplementary Table 1 Oligonucleotide sequences used for PCR amplification and genomic sequencing

Primer Name	Nucleotide sequence(5′ →3′)	Amplicon (bp)	Location[a]	Annealing Temperature (℃)
BNV-F3	GTGAATGAAGACTTTGACG	533	1-533	45
BNV-R3	GCTGACAGGCTCTGGACT			
BNV-F4	CTATGTTGGTGGTGGGAAGAC	862	477-1038	53
BNV-R4	GTAGGCTAGGGTGGACTGG			
BNV-F5	ACAGGGCAGTCCCAGAAT	917	734-1650	52
BNV-R5	GCCGTAGTCATCCCACAA			
BNV-F6	CAAGYTGGCAGAACGCATYG	797	1512-2308	52
BNV-R6	TCCCGTCAGATTGGAGTTCATA			
BNV-F7	GGTAGCCAATAAGCTGAAGG	1293	2184-3476	50
BNV-R7	CCAAAGACCACCCACTCG			
BNV-F8	CARGGCAAGGTCATCAAYG	1180	3340-4519	52
BNV-R8	CTTCCGAAAGGGCACAGA			
BNV-F9	GGCACCGCATTTGTTGGA	804	3946-4749	54
BNV-R9	CTTGGCGTCGTGCGAGAT			
BNV-F10	CCCCTGGCTTTGATRTTT	1139	5424-6562	48
BNV-R10	CACGGGCCGAAGYTGATA			
BNV-F11	ACCTTGCTGTTCTTTATGTGC	1003	6266-7268	47
BNV-R11	AAAACGYCTRCCAMKGTT			

[a] The primer sequences were located at genome sequences of Bo/GIII.2/Adam/2006/No (GenBank accession number JX145650) and Bo/Jena/80/DE (GenBank accession number AJ011099), respectively.

Supplementary Table 2 Oligonucleotide sequences used for verifying genomic sequences

Primer Name	Nucleotide sequence(5′ →3′)	Amplicon (bp)	Location[a]	Annealing Temperature (℃)
BNV-F12	GTGAATGAAGACTTTGACG	440	1-440	49
BNV-R12	TAATGGAAGATGTAACCCT			
BNV-F13	CCAGTTGAACAGCGGAGTA	1113	355-1467	51.6
BNV-R13	GCGCACCCGATTAGACAT			
BNV-F14	CCATCTTGACAGCGTTGAA	1221	788-2008	49.4
BNV-R14	CCTTACCATAGGGAGTGTTGC			
BNV-F15	GATTTCACCCATCTCCAGC	1066	1933-2998	51.2
BNV-R15	CCTCATTGTAGTCAACCTCCC			
BNV-F16	GGGGTGGCAACTATTCTATT	1318	2777-4094	49
BNV-R16	CGCTTCTTCTGAACCTCGT			
BNV-F17	CTCATCAAACGCCCAACA	1240	3268-4507	50
BNV-R17	CTTCCGAAAGGGCACAGA			
BNV-F18	GTCCCAGGTGAACTCCATT	739	4464-5202	51.5
BNV-R18	GGGTTGACTTGTCCAGCA			
BNV-F19	ACTGCTGGACAAGTCAACCC	1178	5183-6360	53.5

BNV-R19	GGCATCAGACACGGAAGG			
BNV-F20	ACGTTCTTGGAGCAGAGTTT	1011	6228-7238	49
BNV-R20	CAATGGCATCCGATCAGTA			

[a] The primer sequences were located at genome sequences of Bo/BET–17/18/CH (GenBank accession number MK159169)

Supplementary Table 3 The RdRp genotype of 43 BNoV-positive samples and co-infection in per region

Provinces	Enteric pathogens	Numbers of samples	GenBank accession number
Sichuan	BNoV(GIII.2) only	2	MK159141, MK131009
	BNoV(GIII.2)+ BRV+ BCoV	4	MK159142, MK159143, MK159146 MK159147
	BNoV(GIII.2)+ BRV+ BVDV	2	MK159144, MK159145
Liaoning	BNoV(GIII.2)+ BRV	2	MK159129, MK130993
	BNoV(GIII.1)+ BRV+ BCoV	2	MK130998, MK130996
	BNoV(GIII.2)+ BRV+ BCoV	12	MK159175, MK159169, MK159135 MK159137, MK159128, MK159130 MK159131, MK159133, MK159134 MK130994, MK13099, MK159132
	BNoV(GIII.2)+ BCoV+ BVDV	1	MK131002
	BNoV(GIII.1)+ BRV+ BCoV+ BVDV	3	MK159136, MK130999, MK131000
	BNoV(GIII.2)+ BRV+ BCoV+ BVDV	5	MK130997, MK131001, MK131004 MK131003, MK159138
Henan	BNoV(GIII.2) only	1	MK159151
	BNoV(GIII.2)+ BRV	1	MK159150
	BNoV(GIII.2)+ BRV+ BCoV	1	MK159149
Shandong	BNoV(GIII.2)+ BRV	1	MK159148
Xinjiang	BNoV(GIII.2) only	2	MK131007, MK131008
	BNoV(GIII.2)+ BRV+ BCoV	1	MK131006
	BNoV(GIII.2)+ BRV+ BVDV	3	MK131005, MK159141, MK159140
